# Explainable Machine Learning for Predicting Adverse Drug Events in Older Adults with Polypharmacy: A Single-Center Retrospective Cohort Study

**DOI:** 10.3390/jcm15155824

**Published:** 2026-07-25

**Authors:** Yun-A Kim, Yoon Jeong Cho, Jonghae Kim, Young Hun Lee, Sang Gyu Kwak

**Affiliations:** 1 Department of Family Medicine, Daegu Catholic University School of Medicine, Daegu 42472, Republic of Korea; yakim@cu.ac.kr (Y.-A.K.); alpha1229@cu.ac.kr (Y.J.C.); 2 Department of Anesthesiology and Pain Medicine, Daegu Catholic University School of Medicine, Daegu 42472, Republic of Korea; usmed@cu.ac.kr (J.K.); david4598@naver.com (Y.H.L.); 3 Department of Medical Statistics, Daegu Catholic University School of Medicine, Daegu 42472, Republic of Korea

**Keywords:** adverse drug event, machine learning algorithms, medication safety, older adults, polypharmacy, Shapley Additive Explanations

## Abstract

**Background:** Polypharmacy is associated with increased adverse drug event (ADE) risk in older adults, but accurate risk stratification remains challenging. This study aimed to develop and evaluate explainable machine learning (ML) models for predicting ADEs in older adults with polypharmacy. **Methods:** This single-center retrospective cohort study included adults aged ≥65 years who received outpatient care at Daegu Catholic University Medical Center between January 2016 and December 2025. Logistic regression, random forest, and Light Gradient-Boosting Machine (LightGBM) models were developed using demographic, comorbidity, medication, and laboratory variables. Model performance was evaluated using discrimination, calibration, and classification metrics. SHapley Additive exPlanations (SHAP) analyses were performed to improve model interpretability. **Results:** A total of 7505 older adults were included, including 366 patients who developed ADEs within 90 days. In the independent test set, random forest demonstrated favorable overall classification performance, achieving the highest accuracy (0.810), specificity (0.828), and F1-score (0.193), whereas logistic regression showed the highest AUROC (0.705) and sensitivity (0.589). SHAP analyses identified medication count, sodium level, diabetes mellitus, and comorbidity burden as major contributors to ADE risk prediction. **Conclusions:** Explainable ML models demonstrated moderate but clinically meaningful performance for predicting ADEs in older adults with polypharmacy. These findings suggest that explainable ML approaches may support clinically interpretable medication safety risk stratification in real-world clinical practice.

## 1. Introduction

Polypharmacy has emerged as a major healthcare challenge in rapidly aging societies because of increasing multimorbidity and the growing complexity of chronic disease management in older adults [1,2,3,4]. As medication burden increases, older individuals become increasingly vulnerable to adverse drug events (ADEs), drug–drug interactions, functional decline, hospitalization, and mortality [2,4,5]. ADEs are particularly important because they represent a common yet potentially preventable source of morbidity and healthcare utilization among older adults [6,7,8,9]. Previous epidemiologic studies have consistently demonstrated that medication burden, comorbidity, and impaired physiologic reserve substantially contribute to medication-related harm in geriatric populations [8,9,10]. In addition, interventions aimed at optimizing polypharmacy remain clinically challenging despite increasing awareness of medication safety in older adults [11].

Early identification of older adults at high risk for ADEs remains difficult in real-world clinical practice. Conventional statistical approaches and existing risk scores have primarily focused on predefined linear associations between clinical variables and adverse outcomes [10,12]. However, ADEs in older adults are often influenced by complex interactions among age-related physiologic changes, multimorbidity, hepatic and renal dysfunction, and cumulative medication exposure. Such multidimensional relationships may not be adequately captured using traditional modeling strategies alone. Recent advances in machine learning (ML) have enabled more flexible predictive modeling capable of identifying nonlinear relationships and complex feature interactions within healthcare datasets [13,14]. Consequently, ML-based approaches have increasingly been explored in medication safety research and ADE prediction using electronic health records [15].

Despite growing interest in artificial intelligence for medication management, several important limitations remain. First, many previous ML studies have primarily focused on potentially inappropriate medications, drug-specific adverse reactions, or imaging-based prediction tasks rather than overall ADE risk stratification in older adults with polypharmacy [15,16,17]. Second, the clinical applicability of many ML models remains limited because of poor interpretability and “black-box” decision-making processes [18]. Third, relatively few studies have simultaneously integrated explainable artificial intelligence techniques with clinically relevant geriatric polypharmacy prediction frameworks [19,20]. Moreover, previous studies have frequently emphasized predictive performance alone without sufficiently evaluating calibration performance, clinical utility, or the relative contribution of clinically relevant predictors to model decision-making.

To address these gaps, we developed and evaluated multiple explainable ML models for predicting ADEs in older adults with polypharmacy using electronic medical record (EMR) data from a tertiary academic medical center. Unlike previous studies that primarily focused on predictive discrimination alone, the present study additionally incorporated SHapley Additive exPlanations (SHAP)-based interpretability analyses to quantify the relative contribution of medication burden, comorbidity burden, and laboratory findings, including renal function parameters such as serum creatinine and estimated glomerular filtration rate (eGFR), to ADE risk prediction [21]. Furthermore, we comprehensively evaluated model discrimination, calibration, and clinical utility using independent test-set validation and decision curve analysis (DCA) [22,23,24]. Through this approach, we aimed to develop clinically interpretable and methodologically robust ML models for medication safety risk stratification in older adults with polypharmacy.

## 2. Materials and Methods

### 2.1. Study Design

This retrospective observational study was conducted using EMR data from Daegu Catholic University Medical Center between 1 January 2016 and 31 December 2025. Demographic information, medication records, comorbidities, and laboratory findings were extracted from the institutional EMR database for ML model development and evaluation. The study period was selected to ensure sufficient longitudinal EMR accumulation and an adequate number of ADEs for model development. Only clinically diagnosed diabetes mellitus and hypertension documented in the EMR database were included because undiagnosed conditions and pre-diabetes could not be reliably identified in this retrospective dataset.

This study was approved by the Institutional Review Board of Daegu Catholic University Medical Center (approval number: DCUMC 2026-03-002; approval date: 11 March 2026). The requirement for informed consent was waived because of the retrospective study design.

### 2.2. Study Population

Older adults aged 65 years or older who received outpatient care and had available medication and laboratory data during the study period were eligible for inclusion. Patients were included if complete medication records and baseline laboratory data were available at the index date. Patients with incomplete clinical records, missing key laboratory variables, duplicate encounters, or inadequate 90-day follow-up data were excluded from the analysis. Complete-case analysis was performed because the overall proportion of missing data was low.

Patients were categorized according to medication burden into three groups: non-polypharmacy (0–4 medications), polypharmacy (5–9 medications), and hyper-polypharmacy (≥10 medications) [25,26,27]. Polypharmacy categories were defined based on the total number of concurrently prescribed medications at the index date.

The following variables were collected from the EMR database: age, sex, body mass index (BMI), Charlson Comorbidity Index (CCI), medication count, hypertension, diabetes mellitus, chronic kidney disease, cardiovascular disease, serum creatinine, eGFR, sodium, potassium, and glucose levels. CCI was selected as a standardized and validated measure of overall comorbidity burden commonly used in prediction modeling studies involving older adults. Hypertension, diabetes mellitus, chronic kidney disease, and cardiovascular disease were identified using ICD-10 diagnostic codes recorded in the EMR database. Medication count was incorporated into ML models as a continuous predictor variable. Laboratory variables were obtained from measurements recorded closest to the index date.

### 2.3. Primary Outcome

The primary outcome was the occurrence of ADEs within 90 days after the index date. ADEs were identified using medication-related ICD-10 diagnostic codes, emergency department visits, hospitalization records, and physician-documented adverse medication-related events within the EMR system. Operational definitions included medication-related diagnostic codes, clinically documented adverse medication reactions, emergency department visits related to medication complications, and hospitalization records attributable to medication-related harm. ADE identification was based on structured electronic medical record–based operational definitions derived from previous medication safety studies and institutional clinical documentation criteria. Detailed operational definitions of ADE-related outcomes are provided in Supplementary Table S1.

### 2.4. ML Model Development

Three ML models were developed and evaluated: logistic regression, random forest, and Light Gradient-Boosting Machine (LightGBM). Logistic regression was included as a conventional interpretable baseline model, while tree-based ensemble methods were selected because of their ability to capture nonlinear relationships and complex interactions among clinical variables.

The dataset was randomly divided into training and independent test sets using an 80:20 ratio with stratification according to ADE occurrence. Because ADE occurrence represented a relatively imbalanced outcome, stratified sampling was applied during dataset partitioning and cross-validation procedures. Additional oversampling, undersampling, or synthetic resampling techniques, such as SMOTE, were not applied to avoid potential overfitting and distortion of clinically observed event distributions. Model development and internal validation were performed exclusively within the training dataset. Internal validation was conducted using stratified five-fold cross-validation to reduce overfitting and evaluate model robustness.

Categorical variables were transformed using one-hot encoding, and continuous variables were standardized for logistic regression analysis when appropriate. Hyperparameters were selected based on prior clinical ML studies and empirically optimized using cross-validation performance while minimizing overfitting. Final hyperparameters were determined according to overall discrimination performance and model stability across validation folds. All analyses were performed using a fixed random seed (2026) to ensure reproducibility. The selected hyperparameters were applied consistently across five-fold cross-validation analyses (Supplementary Table S2).

### 2.5. Model Evaluation

Model performance was comprehensively evaluated using discrimination, calibration, classification, and clinical utility metrics. Discrimination performance was assessed using the area under the receiver operating characteristic curve (AUROC) and the area under the precision–recall curve (AUPRC). Because ADE occurrence represented a relatively imbalanced outcome, AUPRC was additionally evaluated to better assess predictive performance under class imbalance conditions [15].

Classification performance metrics included accuracy, sensitivity, specificity, precision, and F1-score. Sensitivity and F1-score were specifically considered important because accurate identification of high-risk patients is clinically relevant for medication safety surveillance in older adults. Calibration performance was assessed using calibration plots, calibration intercepts and slopes, and the Brier score [22,23]. Lower Brier scores indicated better agreement between predicted probabilities and observed outcomes.

Clinical utility was additionally evaluated using DCA, which estimates the net clinical benefit of prediction models across a range of threshold probabilities [24]. Classification metrics were calculated using a default probability threshold of 0.5. Model performance in the independent test set was additionally evaluated to assess generalizability and robustness of the developed models.

### 2.6. Explainability Analysis

To improve model interpretability, SHAP analyses were performed for the random forest model, which demonstrated slightly superior overall predictive performance in the independent test set [21]. SHAP values were used to quantify the contribution of individual variables to model predictions, thereby enabling interpretation of clinically relevant factors influencing ADE risk prediction. Feature importance was summarized using mean absolute SHAP values, and SHAP summary plots were generated to visualize the direction and magnitude of variable effects on predicted ADE risk. Additional feature importance visualization based on SHAP values was provided to facilitate interpretation of model behavior and clinically important predictors.

### 2.7. Statistical Analysis

No formal sample size calculation was performed because all eligible patients available within the predefined study period were included. Continuous variables are presented as mean ± standard deviation, while categorical variables are presented as number and percentage. Group comparisons were performed using one-way analysis of variance for continuous variables and the chi-square test for categorical variables. All ML analyses were performed using Python (version 3.12) with scikit-learn, LightGBM, and SHAP libraries. A two-sided *p*-value < 0.05 was considered statistically significant. This study was conducted and reported in accordance with the TRIPOD-AI reporting guideline for prediction model development studies.

### 2.8. Generative AI Usage

Generative AI (ChatGPT, GPT-5.5; OpenAI) was used for language editing and clarity improvement. The tool assisted in grammar polishing and stylistic refinement of the manuscript text. No AI tools were used for data analysis, interpretation of results, or scientific decision-making. All scientific content, study design, and conclusions were developed entirely by the authors, who take full responsibility for the manuscript.

## 3. Results

### 3.1. Baseline Characteristics

**Figure 1:**
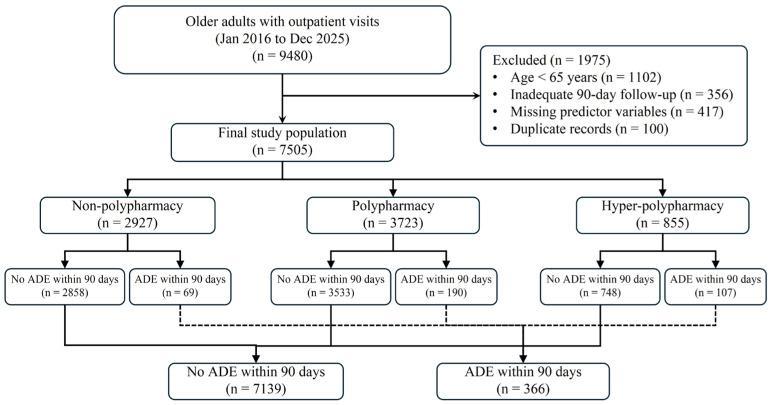
Study flowchart. Older adults with outpatient visits at Daegu Catholic University Medical Center between January 2016 and December 2025 were screened for eligibility. Patients were categorized according to the occurrence of adverse drug events within 90 days. ADE, adverse drug event.

The study flowchart is presented in Figure 1. A total of 7505 older adults were included in the final analysis, including 7139 patients without ADEs and 366 patients with ADEs within 90 days. Baseline characteristics according to polypharmacy status are summarized in Table 1. According to medication burden, 2927 patients (39.0%) were classified into the non-polypharmacy group, 3723 patients (49.6%) into the polypharmacy group, and 855 patients (11.4%) into the hyper-polypharmacy group. Patients with greater medication burden were older and had higher CCI values. Patients with greater medication burden were generally older and had substantially higher comorbidity burden. Age and CCI values progressively increased across the non-polypharmacy, polypharmacy, and hyper-polypharmacy groups (both *p* < 0.001).

The prevalence of major comorbidities progressively increased with greater medication burden. Higher prevalences of hypertension, diabetes mellitus, chronic kidney disease, and cardiovascular disease were consistently observed across increasing polypharmacy categories (all *p* < 0.001). Laboratory findings suggested worse renal function among patients with greater medication burden. Serum creatinine levels increased, whereas eGFR values decreased across polypharmacy categories (both *p* < 0.001). Patients with hyper-polypharmacy additionally showed higher potassium and glucose levels compared with the other groups. In contrast, sodium levels were relatively similar across medication burden categories.

Importantly, higher rates of adverse clinical outcomes were observed in patients with greater medication burden. The incidence of ADEs within 90 days increased from 2.4% in the non-polypharmacy group to 12.5% in the hyper-polypharmacy group (*p* < 0.001). Higher rates of emergency room visits and hospitalization within 90 days were observed in patients with greater medication burden (both *p* < 0.001).

### 3.2. Predictive Performance of ML Models

The predictive performance of the ML models is summarized in Table 2. During five-fold cross-validation, random forest demonstrated slightly superior overall classification performance, achieving the highest F1-score (0.179 ± 0.039) and specificity (0.808 ± 0.016). Logistic regression showed comparable discrimination performance (AUROC: 0.709 ± 0.054) and the highest sensitivity (0.599 ± 0.136), whereas LightGBM demonstrated moderate predictive performance. Detailed fold-specific cross-validation results are provided in Supplementary Table S3.

In the independent test set, random forest demonstrated favorable overall classification performance compared with the other evaluated models, achieving the highest accuracy, specificity, precision, and F1-score. Logistic regression demonstrated the highest AUROC (0.705) and sensitivity (0.589). LightGBM showed moderate predictive performance across discrimination and classification metrics.

**Figure 2:**
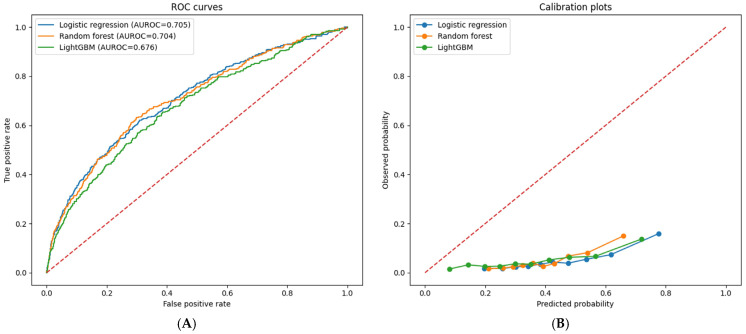
Discrimination and calibration performance of ML models for ADE prediction. (**A**) Receiver operating characteristic curves for LightGBM, logistic regression, and random forest models. The diagonal dashed line represents chance-level discrimination. (**B**) Calibration plots comparing predicted probabilities and observed event probabilities across the evaluated ML models. The diagonal dashed line represents perfect calibration. AUROC, area under the receiver operating characteristic curve.

Receiver operating characteristic curves for all ML models are presented in Figure 2A. Precision–recall curves are additionally presented in Supplementary Figure S1. Calibration plots demonstrated acceptable agreement between predicted and observed probabilities across the evaluated models (Figure 2B). Calibration intercepts and slopes are additionally presented in Supplementary Table S4. DCA additionally suggested favorable clinical utility for the random forest and logistic regression models across clinically relevant threshold probabilities (Supplementary Figure S2).

### 3.3. Logistic Regression Analysis

Multivariable logistic regression analysis identified several independent predictors of ADEs (Table 3). Diabetes mellitus, chronic kidney disease, cardiovascular disease, higher CCI, greater medication burden, and low sodium levels were independently associated with increased ADE risk.

Medication count was also significantly associated with ADE occurrence (OR: 1.095, 95% CI: 1.054–1.138, *p* < 0.001), indicating that increasing medication burden contributed substantially to ADE risk. Among laboratory variables, lower sodium levels were independently associated with increased ADE risk (OR: 0.928, 95% CI: 0.893–0.966, *p* < 0.001). Although sodium levels were relatively similar across polypharmacy categories in the baseline analysis, lower sodium levels remained independently associated with ADE occurrence after multivariable adjustment. In contrast, age, BMI, serum creatinine, eGFR, potassium, and glucose levels were not independently associated with ADE occurrence after adjustment for other clinical variables.

### 3.4. SHAP-Based Model Interpretation

Feature importance derived from SHAP analyses is summarized in Table 4. Medication count was identified as the most influential predictor for ADE prediction, followed by sodium level, diabetes mellitus, CCI, and hypertension. Renal function-related variables, including serum creatinine and eGFR, as well as the presence of chronic kidney disease, also contributed substantially to model prediction performance.

**Figure 3:**
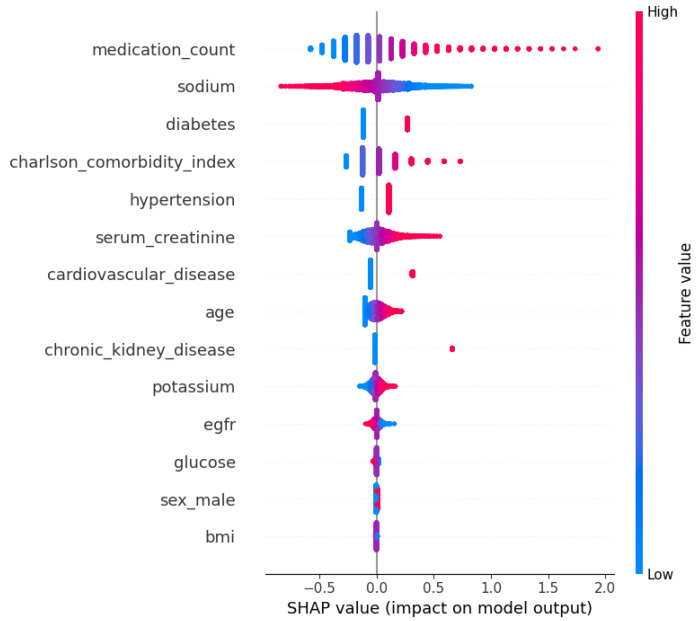
SHAP summary plot for prediction of ADEs. The SHAP summary plot illustrates the contribution of individual variables to model prediction. Each point represents an individual patient. Higher SHAP values indicate a greater contribution to predicted adverse drug event risk. Red and blue colors indicate higher and lower feature values, respectively. SHAP, SHapley Additive exPlanations.

The SHAP summary plot for the random forest model is presented in Figure 3. Higher medication count, greater comorbidity burden, impaired renal function, and low sodium levels were associated with increased predicted risk of ADEs. These findings demonstrated that both medication-related and physiologic variables contributed meaningfully to ADE risk prediction in older adults with polypharmacy. Additional feature importance visualization based on mean absolute SHAP values is presented in Supplementary Figure S3.

## 4. Discussion

In this study, we developed and evaluated multiple explainable ML models for predicting ADEs in older adults with polypharmacy using EMR data from a tertiary academic medical center. Among the evaluated models, random forest demonstrated slightly superior overall predictive performance, whereas logistic regression showed comparable discrimination ability with higher sensitivity and interpretability. In addition, SHAP-based explainability analyses identified medication burden, sodium level, comorbidity burden, and renal function–related variables as major contributors to ADE risk prediction.

The present findings reinforce the well-established relationship between polypharmacy and medication-related harm in older adults. Previous studies have consistently demonstrated that increasing medication burden is associated with higher risks of ADEs, hospitalization, and mortality [2,3,4,5]. In our study, the incidence of ADEs increased substantially across polypharmacy categories, particularly among patients with hyper-polypharmacy. Medication count was also identified as the most influential predictor in SHAP analyses and remained independently associated with ADE occurrence in multivariable logistic regression analysis. These findings support the concept that cumulative medication exposure itself represents a major determinant of medication-related vulnerability in older adults.

Comorbidity burden and renal dysfunction also emerged as important contributors to ADE risk prediction. Chronic kidney disease and CCI were identified as clinically relevant predictors in multivariable regression, while renal function–related variables such as serum creatinine and eGFR also contributed to model predictions in SHAP analyses. Older adults with impaired renal function are particularly susceptible to altered pharmacokinetics, drug accumulation, and medication toxicity, which may increase the likelihood of ADEs [8,9]. Furthermore, although baseline sodium levels were relatively similar across polypharmacy categories, lower sodium levels independently contributed to ADE risk prediction after multivariable adjustment. This finding may reflect underlying frailty, chronic illness burden, or medication-related electrolyte disturbances commonly observed in older adults receiving multiple medications [28,29].

An important strength of the present study is the incorporation of explainable artificial intelligence techniques into ADE prediction modeling. Although ML methods have increasingly been explored in medication safety research [15], clinical implementation remains challenging because many ML algorithms function as “black-box” systems with limited interpretability [18]. To address this limitation, we used SHAP analyses to quantify the relative contribution of individual variables to model predictions. This approach enabled visualization of clinically plausible relationships between medication burden, lower sodium levels, renal dysfunction, comorbidity burden, and ADE risk. Such interpretability may improve clinician trust and facilitate the integration of ML-based prediction tools into real-world medication management strategies.

Interestingly, logistic regression demonstrated predictive performance comparable to that of more complex ML models. This finding is consistent with previous studies suggesting that traditional statistical approaches may remain competitive with advanced ML methods in certain clinical prediction tasks involving structured tabular healthcare data [16]. Although random forest achieved slightly superior overall predictive performance in our study, the relatively similar performance of logistic regression highlights the importance of balancing predictive accuracy with model interpretability and clinical usability. Nevertheless, despite comparable discrimination performance, tree-based ML models may still offer practical advantages by capturing complex nonlinear relationships and interactions among clinical variables without requiring prespecified interaction structures. In addition, SHAP-based explainability analyses enabled intuitive visualization of individualized predictor contributions, which may facilitate integration into EMR-based clinical decision support systems.

The discrimination performance observed in the present study was moderate; however, previous ADE prediction studies using routinely collected healthcare data have similarly reported AUROC values in the range of approximately 0.65–0.75 [15]. Therefore, the present models may still provide clinically meaningful risk stratification when integrated into EMR-based medication safety screening systems in conjunction with clinical review. The relatively low precision and F1-scores observed across the evaluated models likely reflect the low overall incidence of ADEs in the study population. In medication safety screening, however, higher false-positive rates may be clinically acceptable because the primary goal is early identification of potentially vulnerable patients who may benefit from additional medication review and monitoring.

Although the overall discrimination was moderate (AUROC approximately 0.70), the intended role of this model is not to replace clinical judgment but to support medication safety screening in routine outpatient practice. During outpatient visits, the model could automatically estimate an individual patient’s 90-day ADE risk using routinely available demographic characteristics, comorbidities, medication burden, and laboratory results. Patients identified as high risk could then undergo targeted medication reconciliation, pharmacist-led medication review, evaluation of potentially inappropriate medications, dose adjustment according to renal function, correction of reversible electrolyte abnormalities, and closer follow-up after prescription changes. Conversely, patients classified as low risk may avoid unnecessary intensive monitoring, allowing more efficient allocation of clinical resources.

Such a risk-guided strategy may facilitate earlier identification of vulnerable older adults before clinically significant ADEs occur. Rather than relying solely on medication count, clinicians could prioritize patients according to individualized predicted risk, thereby improving medication safety while reducing preventable emergency department visits, hospitalizations, and medication-related healthcare utilization. Because all predictor variables are routinely collected in electronic medical records, implementation as an automated clinical decision support tool would require minimal additional workload for clinicians.

The present study has several limitations. First, this was a retrospective single-center study, which may limit generalizability to other healthcare systems or patient populations. Second, ADE identification was based on EMR-derived clinical documentation and administrative records, which may have resulted in under-recognition of mild or undocumented ADEs. Third, despite the inclusion of multiple clinically relevant variables, residual confounding and unmeasured factors may still have influenced prediction performance. Fourth, external validation using independent multicenter datasets was not performed. Future studies incorporating prospective validation, multicenter external validation, and temporal medication exposure patterns are necessary to further improve the generalizability and clinical applicability of these models before routine clinical implementation.

## 5. Conclusions

Explainable ML models demonstrated moderate but clinically meaningful performance for predicting ADEs in older adults with polypharmacy. Among the evaluated models, random forest showed slightly superior overall predictive performance, whereas logistic regression demonstrated comparable discrimination ability with higher sensitivity and interpretability. Medication burden, sodium level, comorbidity burden, and renal function–related variables were identified as major contributors to ADE risk through SHAP-based explainability analyses. These findings suggest that explainable machine learning models may serve as automated medication safety screening tools for older adults with polypharmacy. By identifying patients who are most likely to benefit from comprehensive medication review, pharmacist intervention, individualized dose adjustment, and closer follow-up, these models may support safer prescribing practices and more efficient allocation of healthcare resources. Future prospective and multicenter validation studies are warranted before routine clinical implementation. Such approaches may additionally assist clinicians in identifying potentially modifiable factors contributing to medication-related harm risk.

## Figures and Tables

**Table 1:** Baseline characteristics according to polypharmacy status.

Variable	Non-Polypharmacy (*n* = 2927)	Polypharmacy (*n* = 3723)	Hyper-Polypharmacy (*n* = 855)	*p*-Value
Age	72.55 ± 5.51	74.90 ± 6.03	78.44 ± 6.41	<0.001
BMI	24.11 ± 3.19	24.15 ± 3.16	24.43 ± 3.14	0.039
CCI	1.25 ± 1.00	1.88 ± 1.09	3.05 ± 1.26	<0.001
Medication count	2.90 ± 1.06	6.54 ± 1.31	11.96 ± 2.34	<0.001
Serum creatinine	0.80 ± 0.20	0.84 ± 0.21	0.95 ± 0.25	<0.001
eGFR	85.09 ± 13.07	81.49 ± 13.39	74.83 ± 14.34	<0.001
Sodium	139.94 ± 2.88	140.02 ± 2.90	139.90 ± 2.75	0.400
Potassium	4.22 ± 0.36	4.24 ± 0.35	4.29 ± 0.37	<0.001
Glucose	109.64 ± 26.01	114.85 ± 27.67	121.96 ± 27.70	<0.001
Male sex	1286 (43.9)	1666 (44.7)	350 (40.9)	0.128
Hypertension	1231 (42.1)	2293 (61.6)	692 (80.9)	<0.001
Diabetes mellitus	495 (16.9)	1251 (33.6)	486 (56.8)	<0.001
Chronic kidney disease	28 (1.0)	141 (3.8)	175 (20.5)	<0.001
Cardiovascular disease	131 (4.5)	461 (12.4)	283 (33.1)	<0.001
ADE within 90 days	69 (2.4)	190 (5.1)	107 (12.5)	<0.001
ER visit within 90 days	209 (7.1)	331 (8.9)	106 (12.4)	<0.001
Hospitalization within 90 days	109 (3.7)	175 (4.7)	77 (9.0)	<0.001

Data are presented as mean ± standard deviation or number (%). *p*-values were calculated using one-way analysis of variance or the chi-square test, as appropriate. Non-polypharmacy was defined as the concurrent use of 0–4 medications, polypharmacy as 5–9 medications, and hyper-polypharmacy as ≥10 medications. ADE, adverse drug event; BMI, body mass index; CCI, Charlson comorbidity index; eGFR, estimated glomerular filtration rate; ER, emergency room.

**Table 2:** Predictive performance of ML models for ADE prediction.

Model	LightGBM	Logistic Regression	Random Forest
Five-fold cross validation			
AUROC	0.677 ± 0.032	0.709 ± 0.054	0.708 ± 0.047
AUPRC	0.130 ± 0.020	0.154 ± 0.024	0.151 ± 0.029
Accuracy	0.771 ± 0.008	0.701 ± 0.015	0.791 ± 0.011
Sensitivity	0.443 ± 0.065	0.599 ± 0.136	0.473 ± 0.132
Specificity	0.788 ± 0.011	0.706 ± 0.020	0.808 ± 0.016
Precision	0.096 ± 0.009	0.094 ± 0.015	0.111 ± 0.022
F1-score	0.158 ± 0.017	0.162 ± 0.028	0.179 ± 0.039
Brier score	0.161 ± 0.004	0.208 ± 0.007	0.173 ± 0.004
Independent test set			
AUROC (95% CI)	0.676 (0.603–0.748)	0.705 (0.634–0.776)	0.704 (0.632–0.775)
AUPRC	0.141	0.174	0.157
Accuracy	0.771	0.710	0.810
Sensitivity	0.466	0.589	0.466
Specificity	0.786	0.716	0.828
Precision	0.100	0.096	0.121
F1-score	0.165	0.165	0.193
Brier score	0.163	0.204	0.170

Data are presented as mean ± standard deviation across five-fold cross-validation analyses. Performance metrics in the independent test set were evaluated using a held-out test dataset comprising 20% of the total study population. Ninety-five percent confidence intervals for AUROC values were estimated using bootstrap resampling. Model performance was assessed using the AUROC, AUPRC, accuracy, sensitivity, specificity, precision, F1-score, and Brier score. AUROC, area under the receiver operating characteristic curve; AUPRC, area under the precision–recall curve.

**Table 3:** Multivariable logistic regression analysis for prediction of ADEs.

Variable	OR	Lower 95% CI	Upper 95% CI	*p*-Value
Age	1.014	0.993	1.035	0.192
Male sex	0.925	0.736	1.164	0.508
BMI	1.004	0.969	1.041	0.826
Hypertension	1.267	0.968	1.659	0.084
Diabetes mellitus	1.365	1.024	1.820	0.034
Chronic kidney disease	1.700	1.039	2.781	0.035
Cardiovascular disease	1.361	1.002	1.848	0.049
CCI	1.175	1.040	1.328	0.010
Medication count	1.095	1.054	1.138	<0.001
Serum creatinine	1.644	0.897	3.012	0.108
eGFR	0.995	0.986	1.004	0.292
Sodium	0.928	0.893	0.966	<0.001
Potassium	1.125	0.821	1.541	0.465
Glucose	1.000	0.995	1.005	0.953

Odds ratios (ORs) and 95% confidence intervals (CIs) were estimated using multivariable logistic regression analysis. The dependent variable was ADE occurrence within 90 days. BMI, body mass index; CCI, Charlson comorbidity index; CI, confidence interval; eGFR, estimated glomerular filtration rate; OR, odds ratio.

**Table 4:** Top variables contributing to ADE prediction based on SHAP values.

Rank	Variable	Mean Absolute SHAP Value
1	Medication count	0.2429
2	Low sodium	0.1748
3	Diabetes mellitus	0.1618
4	CCI	0.1368
5	Hypertension	0.1179
6	High serum creatinine	0.0897
7	Cardiovascular disease	0.0818
8	Age	0.0536
9	Chronic kidney disease	0.0432
10	Potassium	0.0318
11	Low eGFR	0.0216
12	Glucose	0.0069
13	Male sex	0.0040
14	BMI	0.0017

Feature importance was quantified using the mean absolute SHAP values derived from the random forest model. Higher SHAP values indicate greater contribution to model prediction. BMI, body mass index; CCI, Charlson comorbidity index; eGFR, estimated glomerular filtration rate; SHAP, SHapley Additive exPlanations.

## Data Availability

The data presented in this study are available on request from the corresponding author under ethical and legal restrictions related to patient confidentiality. The analysis code used in this study is available from the corresponding author upon reasonable request.

## Supplementary Materials

The following supporting information can be downloaded at: https://www.mdpi.com/article/10.3390/jcm15155824/s1, Table S1. Operational definitions of adverse drug event-related outcomes; Table S2. Hyperparameters of machine learning models; Table S3. Five-fold cross-validation results for machine learning models; Table S4. Calibration intercepts and slopes of machine learning models; Figure S1. Precision–recall curves of machine learning models for predicting adverse drug events; Figure S2. Decision curve analysis of machine learning models; Figure S3. Feature importance based on mean absolute SHAP values; File S1: TRIPOD-AI Checklist.

## Abbreviations

The following abbreviations are used in this manuscript:

ADE	Adverse drug event
AUPRC	Area under the precision–recall curve
AUROC	Area under the receiver operating characteristic curve
BMI	Body mass index
CCI	Charlson Comorbidity Index
CI	Confidence interval
DCA	Decision curve analysis
eGFR	Estimated glomerular filtration rate
EMR	Electronic medical record
ER	Emergency room
LightGBM	Light Gradient-Boosting Machine
ML	Machine learning
OR	Odds ratio
SHAP	SHapley Additive exPlanations
TRIPOD-AI	Transparent Reporting of a multivariable prediction model for Individual Prognosis Or Diagnosis–Artificial Intelligence
