# Supplementary material for: Explainable Machine Learning for Predicting Adverse Drug Events in Older Adults with Polypharmacy: A Single-Center Retrospective Cohort Study
Source: J Clin Med. 2026 Jul 25;15(15):5824. doi: 10.3390/jcm15155824 (PMC13467294; doi:10.3390/jcm15155824)
Supplement: Supplementary file 1 [file jcm-15-05824-s001.zip › Supplementary.pdf]

Supplementary Materials

Supplementary Tables

Supplementary Table S1. Operational definitions of adverse drug event-related outcomes

ADE category	Operational definition
Hypoglycemia	ICD diagnosis or documented glucose <70 mg/dL requiring clinical evaluation or medical attention
Acute kidney injury	Increase in serum creatinine according to KDIGO criteria
Electrolyte abnormality	Clinically documented sodium or potassium abnormality requiring medical evaluation or treatment
Drug-related hospitalization	Hospital admission associated with medication-related diagnosis
Drug-related ER visit	Emergency department visit associated with medication-related diagnosis

Supplementary Table S2. Hyperparameters of machine learning models

Model	Hyperparameters
Logistic regression	max_iter=1000;<br>class_weight=balanced;<br>solver=lbfgs
Random forest	n_estimators=500;<br>max_depth=5;<br>min_samples_leaf=20;<br>class_weight=balanced;<br>random_state=2026
LightGBM	n_estimators=400;<br>max_depth=3;<br>learning_rate=0.03;<br>subsample=0.8;<br>colsample_bytree=0.8;<br>class_weight=balanced;<br>random_state=2026

Hyperparameters were selected based on prior clinical machine learning studies and optimized empirically to balance discrimination performance and overfitting.

Supplementary Table S3. Five-fold cross-validation results for machine learning models

Model	Fold	AUROC	AUPRC	Accuracy	Sensitivity	Specificity	Precision	F1-score	Brier score
LightGBM	1	0.679	0.124	0.767	0.493	0.781	0.103	0.171	0.165
	2	0.730	0.165	0.769	0.493	0.784	0.104	0.172	0.159
	3	0.665	0.127	0.772	0.466	0.788	0.101	0.166	0.158
	4	0.658	0.126	0.762	0.425	0.779	0.09	0.148	0.166
	5	0.651	0.111	0.783	0.338	0.807	0.083	0.133	0.157
Logistic regression	1	0.746	0.166	0.688	0.671	0.689	0.099	0.173	0.212
	2	0.785	0.181	0.702	0.795	0.697	0.118	0.206	0.212
	3	0.671	0.163	0.700	0.548	0.708	0.088	0.151	0.206
	4	0.662	0.123	0.690	0.534	0.697	0.083	0.143	0.212
	5	0.681	0.135	0.725	0.446	0.739	0.081	0.138	0.197
Random forest	1	0.729	0.157	0.790	0.562	0.802	0.127	0.207	0.175
	2	0.780	0.198	0.792	0.644	0.800	0.141	0.232	0.176
	3	0.686	0.138	0.785	0.452	0.802	0.104	0.17	0.171
	4	0.668	0.122	0.780	0.397	0.800	0.092	0.149	0.176
	5	0.676	0.141	0.810	0.311	0.836	0.089	0.139	0.167

Performance metrics were calculated for each fold during stratified five-fold cross-validation analyses. AUROC and AUPRC were used to evaluate discrimination performance, while the Brier score was used to assess calibration performance. AUROC, area under the receiver operating characteristic curve; AUPRC, area under the precision–recall curve.

Table S4. Calibration intercepts and slopes of machine learning models

Model	Calibration intercept	Calibration slope
Logistic regression	-2.983	1.041
Random forest	-2.625	1.536
LightGBM	-2.669	0.797

A calibration intercept close to 0 and a calibration slope close to 1 indicate better agreement between predicted and observed probabilities.

Supplementary figures legend

Supplementary Figure S1. Precision–recall curves of machine learning models for predicting adverse drug events. Precision–recall curves for LightGBM, logistic regression and random forest models are shown. The area under the precision–recall curve (AUPRC) was used to evaluate predictive performance under class imbalance conditions. AUPRC, area under the precision–recall curve.

Supplementary Figure S2. Decision curve analysis of machine learning models. Decision curve analysis comparing the clinical net benefit of LightGBM, logistic regression, and random forest models across a range of threshold probabilities for adverse drug event prediction. The dashed lines represent the treat-all and treat-none strategies.

Supplementary Figure S3. Feature importance based on mean absolute SHAP values. Feature importance was quantified using the mean absolute SHAP values derived from the best-performing machine learning model. Variables with higher SHAP values contributed more strongly to model prediction of adverse drug events. SHAP, SHapley Additive exPlanations.
